# Everyday Life Experiences of Swedes Age 70+ During the COVID-19 Pandemic

**DOI:** 10.1093/geroni/igab046.079

**Published:** 2021-12-17

**Authors:** Torbjorn Bildtgard, Peter Öberg

**Affiliations:** 1 Stockholms University, Stockholm, Stockholms Lan, Sweden; 2 University of Gävle, Gävle, Gavleborgs Lan, Sweden

## Abstract

As many other countries Sweden has been hit hard by the Corona pandemic, with high numbers of dead in the older population. Since march 16, 2020, the authorities have encouraged people 70+ to voluntarily quarantine and avoid contacts outside the household. How has this affected older people’s everyday lives? This study reports on results from a web-survey on the everyday life experiences of Swedes 70+ carried out between May 28 and July 13, 2020 (n=1 926). The presentation focuses answers to an open-ended question: “Describe with your own words how your life has been affected by the Corona pandemic”. A qualitative content analysis was used to investigate changes in the everyday lives of the respondents and their appreciations of these changes. Results show that older Swedes have mostly adhered to public recommendations of self-isolation and withdrawn from social and family contacts, as well as paid and volunteer work. The vast majority (76%) of the respondents describe what they see as negative life changes, such as loss of structure in their everyday life, loss of contact with children/grandchildren and friends, loss of meaningful activities, loss of abilities due to forced unemployment and experiences of ageism. Experiences of loneliness, depression and drop in quality of life are common. Some positive changes were reported. We argue that the experience of the 70+ population during Corona needs to be understood in relation to the promise of the third age, where everyday restrictions are experienced as a forced disengagement into a fourth age life style.

